# Racial Differences in the Erosion of Psychological Resilience Following COVID-19 Related Financial Hardship

**DOI:** 10.1093/geroni/igab046.358

**Published:** 2021-12-17

**Authors:** Miles Taylor, Dawn Carr, Kendra Jason

**Affiliations:** 1 Florida State University, Tallahassee, Florida, United States; 2 UNC Charlotte, Charlotte, North Carolina, United States

## Abstract

Objectives: Research on the impact of COVID-19 among older adults has primarily focused on virus outcomes, but it is also possible the pandemic’s hardships have eroded the adaptive capacity of older adults. It is also likely these impacts vary by race and ethnicity. We examine changes in psychological resilience (PR) among older adults pre and post-pandemic to determine whether financial and social hardships have altered this resource for White, Black, and Hispanic older adults. Method: Using the COVID module recently released by the HRS (n=735), we examined changes in PR between 2016 and 2020 related to specific COVID experiences. We tested interactions to determine whether the effects of these experiences were conditioned by race and ethnicity. Results: Consistent with previous literature, resilience was relatively stable during this time on average. Financial hardship due to COVID-19 diminished resilience, but this effect was concentrated primarily among White Americans. PR was unchanged related to financial hardship among Black Americans. Discussion: The results suggest that PR is a relatively stable resource in later life, even during a pandemic. However, this resource may be impacted in the face of specific and especially new challenges in later life. Policies and interventions related to job loss and financial hardship during the pandemic should be seen as supporting the capacity for older adults to adapt to current as well as future challenges.

